# Cytotoxicity evaluation and metabolomic profiling of *Spheciospongia vagabunda*-associated fungi corroborated by in silico studies

**DOI:** 10.1038/s41598-025-04162-6

**Published:** 2025-06-20

**Authors:** Omnia Hesham Abdelhafez, Miada F. Abdelwahab, Abeer H. Elmaidomy, Arwa Mortada Ahmed, Mohamed Hisham, Stefanie P. Glaeser, Peter Kämpfer, Jun Wu, Usama Ramadan Abdelmohsen

**Affiliations:** 1 Department of Pharmacognosy, Faculty of Pharmacy, Deraya University, New Minia, 61111 Egypt; 2 Department of Pharmacognosy, Faculty of Pharmacy, Minia University, Minia, 61519 Egypt; 3 Department of Pharmacognosy, Faculty of Pharmacy, Beni-Suef University, Beni-Suef, 62514 Egypt; 4 Department of Pharmaceutical Chemistry, Faculty of Pharmacy, Deraya University, New Minia, 61111 Egypt; 5 Institute of Applied Microbiology, Justus-Liebig University Giessen, 35392 Giessen, Germany; 6 Guangdong Key Laboratory for Research and Development of Natural Drugs, College of Pharmacy, Guangdong Medical University, Dongguan, 523808 China

**Keywords:** Cytotoxicity, *Spheciospongia vagabunda*, *Aspergillus*, *Penicillium*, Metabolomics, Molecular docking, Cancer, Computational biology and bioinformatics, Drug discovery, Microbiology

## Abstract

Cancer incidence continues to increase every year. Scientists strive to search for new anticancer compounds to combat this disease. Appealingly, marine environmental niches are still an untapped scaffold for natural products with chemical and biomedical diversity. Hence, fungi isolated from the Red Sea sponge *Spheciospongia vagabunda* were explored. Two strains were purified from the sponge and identified, depending on 18 S rRNA gene sequence, as *Aspergillus* sp. (UR1) and *Penicillium* sp. (UR2). The obtained fungal extracts were submitted to LC-HR-ESI-MS metabolomics evaluation, which showed notable variation in the chemical profiles of both extracts. The cytotoxic activity was assessed against three cancer cell lines: HepG2 (hepatocellular carcinoma human), MCF7 (breast cancer) and CaCo-2 (human colon carcinoma), *via* MTT assay. UR1 extract displayed higher antiproliferative activity with IC_50_ values 2.61 ± 0.12, 3.23 ± 0.21 and 3.41 ± 0.18 µg/ml against HepG2, CaCo-2 and MCF7, respectively. Whereas UR2 extract exhibited a less potent effect with IC_50_ values of 17.65 ± 0.28, 18.38 ± 0.19, and 22.45 ± 0.27 µg/ml. Additionally, molecular docking was conducted. Most identified compounds established strong binding affinity with the PPARG gene. Compounds **6** and **16** showed binding energy with S values − 9.13 and − 8.38 kcal/mol, respectively. The findings suggested the importance of *Spheciospongia vagabunda*-derived fungi in the production of cytotoxic natural compounds that could be used for cancer management.

**Supplementary Information:**

The online version contains supplementary material available at 10.1038/s41598-025-04162-6.

## Introduction

Cancer is a disease involving impaired cellular function characterized by abnormal cells growing beyond their natural boundaries^1^. It is globally considered a primary public health and among the leading causes of mortality, hence, it puts a tremendous burden on healthcare systems^2^. This threat is estimated to aggravate enormously during the coming years owing to the growing population, socioeconomic effects, and exposure to risk factors including chemicals, radiation, viruses, and genetic mutations^3,4^. Scientists are facing a variety of challenges related to the presently available anticancer medicaments. Accordingly, they are attempting to discover therapeutic alternatives that could be less toxic to patients and more active against resistant tumours^4^.

Numerous in vitro and in vivo investigations confirmed the antitumour potential of natural products^5^. Microbes, plants and marine organisms have all been used to produce anticancer leads^6^. Notably, marine ecological niches have been demonstrated to be a massive reservoir of structurally distinct bioactive metabolic products due to their unexplored biodiversity and harsh atmosphere compared to terrestrial habitats. This chemical diversity correlates exponentially with unexpected pharmacological activities and new mechanisms of action^7^. Marine fungi, derived from marine invertebrates, including sponges and soft corals, as well as fungi from marine plants or marine sediments have been observed to possess profound chemical and biological profiles. Several potent anticancer compounds have been identified from marine-associated fungi and are depicted as potential candidates for medical application^8^. Plinabulin, a fungal product from marine origin, has advanced to clinical studies^9,10^.

Remarkably, fungi constitute up to 73% of sponge-derived microbes. In addition, approximately 20% of all new secondary metabolites from marine fungi were purified from sponge-derived ones between 2010 and 2020^11^. Moreover, sponges have been investigated extensively as massive producers of cytotoxic compounds, and in various cases, the isolated metabolites were found in the fungi isolated from the same sponge^12–15^. Therefore, exploiting marine sponge-derived fungi can, in some cases, boost the production of some valuable metabolites derived from sponges and, in other cases aiming to fight and adapt to the marine environmental conditions, they can biosynthesize completely new metabolites^16^. This fascinating relationship between sponges and their associated fungi has inspired us to study the fungi obtained from the Red Sea-derived sponge *Spheciospongia vagabunda*.

The marine sponge *Spheciospongia vagabunda* belongs to family *Clionaidae*^17^. Extracts obtained from *S. vagabunda* have displayed cytotoxic effects against several tumour cells^18,19^. Subsequent chemical inspection of *S. vagabunda* has brought about the purification of many secondary metabolites. For instance, ceramides that have exhibited pronounced cytotoxicity against liver and breast cancer cell lines^18,20^.

In the current research work, we explore the fungi associated with the Red Sea-derived sponge *S. vagabunda* with regard to the diversity in biology and chemistry. Two fungi were isolated and identified as *Aspergillus* sp. (UR1) and *Penicillium* sp. (UR2). The chemical profiles of these fungi were analysed by means of LC-HRES-MS-based untargeted metabolomics. The extracts derived from each culture were as well in vitro evaluated for the cytotoxic potential against three different cancer cell lines. Eventually, in silico molecular docking was implemented to inspect the most active metabolites and to predict their putative action mechanism. Our findings heightened the relevance of *Spheciospongia vagabunda*-associated fungi as an interesting source for natural anticancer lead molecules, imposing further investigations.

## Materials and methods

###  Collection and identification of the sponge

The sponge was collected from Ahia Reefs, in the Red Sea (at latitudes 27°17′01.0′′ N and longitudes 33°46′21.0′′ E), nearly five kilometers north of Hurghada, at a depth of approximately 3 m. This area is distinguished by a long irregular reef with many lagoons and depressions. The bottom topography is featured by the presence of corals besides the seagrasses and algae in subtidal and intertidal areas. El-Sayed Abde El-Aziz (Invertebrates Department, National Institute of Oceanography and Fisheries, Red Sea Branch, 84511 Hurghada, Egypt), identified the collected sponge as *Spheciospongia vagabunda*. The sponge sample was immediately put in a sterilized plastic bag comprising seawater inside an icebox, then carried for fungal isolation in the laboratory. A voucher specimen has been stored in the Pharmacognosy Department herbarium, Faculty of Pharmacy, Deraya University, New Minya City, Egypt with the number of registration Der-Ph-0112.

### Chemicals

All reagents and chemicals utilized in this current research work were of high analytical grade, they were acquired from Sigma Chemical Co Ltd. (St Louis, MO, USA) and Merck (Germany).

###  Isolation and purification of the sponge-associated fungi

The sponge biomass was washed using sterilized sea water, dissected into fragments of about 1 cm^3^, then rigorously homogenized with nearly 10 times the volume of sterile seawater in a sterile mortar. Sabouraud dextrose agar (SDA) was used as a solid growth medium for isolating of the marine sponge-derived fungal strains. Amoxicillin and gentamycin (100 µg/L) were added to the prepared medium, in order to inhibit any contamination from bacteria. The supernatant was subjected to serial dilutions of 1 × 10^− 2^, 1 × 10^− 3^, 1 × 10^− 6^ and 1 × 10^− 9^, then subsequently transferred into the previously prepared media and set for incubation at 30 °C (1–3 days). The fungal growth was carefully monitored and the distinct hyphal tips were taken away and repeatedly sub-cultured till pure strains were recovered. For long-term storage, pure strains were maintained on plates containing 30% glycerol at -80 °C^5,21^.

### Molecular identification of the isolated fungi and phylogenetic analysis

The fungal strains UR1 and UR2 were identified phylogenetically using sequence analyses of the partial 18 S rRNA gene and ITS (internal transcribed spacer) region, that comprises ITS1, 5.8 S rRNA gene, and ITS2 sequences, along with MasterPure Yeast DNA extraction kit (Epientre, Madison, Wisconsin), which was used to extract DNA from fungal biomass. Employing the universal fungal primers NS1 (5′- GTAGTCATATGCTTGTCTC − 3′)^22^ and ITS-4 (5′-TTCCTCCGCTTATTGATATGC-3′)^23^, DNA amplification of the 18 S rRNA gene and the complete ITS region was carried out. Sanger sequencing was performed with primers NS1 (partial 18 S rRNA gene sequence analysis) and ITS-4 (ITS sequence analysis) by LGC Genomics (Germany). MEGA11 version 11.0.1 was used for phylogenetic analysis and manual sequence corrections^24^. The RefSeq Targeted Loci project databases (BioProjects PRJNA39195 and PRJNA177353; both updated on 2024-10-18) were used to identify next-related strains using the BLASTn tool of NCBI (https://www.ncbi.nlm.nih.gov/). The next-type material strains’ 18 S rRNA gene sequences and ITS sequences have been loaded into MEGA11 and aligned utilizing ClustalW^25^. Uniform rates were applied at all nucleotide sites, and paired deletions were used to compare sequences. The General Time Reversible model^26^ was used for the 18 S rRNA gene and the Kimura 2-parameter model^27^ for the ITS sequence dissimilarity matrix construction to determine phylogenetic trees, along with the maximum likelihood method (18 S rRNA gene sequences) and the neighbour joining method (ITS sequences). The bootstrap option (100 replications) was used to test the phylogenies. For the examination of 18 S rRNA gene and ITS sequence-based analyses, a total of 50 to 100 reference sequences were considered each. Number of reference sequences were further reduced during tree construction. Type material sequences of *Talaromyces* species were used as outgroup sequences. The gene sequences of strains UR1 and UR2 were submitted to GenBank/EMBL/DDBJ with accession numbers PP843230-PP843231 (18 S rRNA gene sequences) and PP843228-PP843229 (ITS sequences).

### Cultivation of the pure fungal strains and culture extraction

The two pure fungal isolates were cultivated on a solid medium^28,29^. About 150 µL of each fungus was transferred to twenty solid plates of SDA (10 g peptone, 40 g dextrose, and 20 g agar in 1 L distilled water, Merk). Following ten days of incubation at 30 °C, agar was cut into tiny parts and then put in flasks with the extraction solvent (ethyl acetate, 300 ml). Ethyl acetate is a suitable extraction solvent because it stops the fermentation and diminishes the spores which could be brought into air after opening the Petri dishes^30,31^. Disruption of the mycelia is accomplished by utilizing the ultrasonic cleaner (Bransonic^®^) at power of 100 W, for at least 30 min. The crude extract (0.5 g) from every fungus was obtained after filtration and concentration with decreased pressure at nearly 40 °C, with the aid of a rotary evaporator (Heidolph^®^, 154 rpm).

###  LC-HR-ESI-MS metabolomics analysis

The obtained ethyl acetate extracts of the two isolated fungi were submitted to LC-HR-ESI-MS metabolomics analysis as previously reported^32,33^. Reversed-phase HPLC column was employed as the stationary phase in this chromatographic separation. It was a HiChrom (Berkshire, UK) C18, 75 mm × 3.0 mm, 5 μm HPLC column. Gradient elution was carried out with the use of purified water (A) and acetonitrile (B) in addition to formic acid (0.1%), applied at 300 µL/min. The chromatographic steps began by 10% B, then gradually raised to reach 100% B, followed by isocratic elution for 5 min, then reduced back to 10% B for one min (Accela HPLC, Thermo Fisher Scientific, Karlsruhe, Germany) using UV-visible detector and mass spectrometer (Exactive-Orbitrap, Thermo Fisher Scientific). The HR-ESI-MS was conducted employing negative and positive ionization modes combined with spray voltage at 4.5 Kv, mass ranging from *m/z* 150 to 1500 and capillary temperature at 320 °C. Mzmine 2.10 data mining software was used to analyze the unprocessed MS data. Dictionary of Natural Products (DNP) http://dnp.chemnetbase.com/faces/chemical/Chemi calSearch.xhtml as well as METLIN were the databases employed for the identification of the dereplicated metabolites. Whereas, the chemical structures were drawn using Chem Bio Draw Ultra 14.0 software.

### Cytotoxicity assay

The cytotoxicity was in vitro tested against several cancer cell lines; comprising hepatocellular carcinoma (HepG2), human breast cancer (MCF7) and human colon carcinoma (CaCo-2), implementing the 3-(4,5-dimethylthiazol-2-yl)-2,5-diphenyltetrazolium bromide (MTT) method^34^. The American Type Culture Collection (Manassas, VA, USA) is where the cell lines were purchased. The cells were cultured utilizing DMEM (Invitrogen/Life Technologies, USA) in addition to 10% FBS (Hyclone, USA), 1% streptomycin-penicillin and 10 µg/mL of insulin (Sigma-Aldrich, Germany), then placed into 96-well plates (at a density of 1.2–1.8 × 10,000 cells/well) in a volume of 100 µL with 100 µL of the tested sample per well, added in concentrations of 100, 25, 6.3, 1.6, 0.4 µg/mL. Then after 24 h, equal amounts of the MTT solution were included with 10% of the volume of the culture medium. Two to four hours were spent for incubation of the cultures, according to cell densities and their metabolic activities, at 37 °C and 5% CO_2_. Afterward, the cultures were taken out of the incubator, then DMSO was added in equal parts to the volume of the original culture medium for the purpose of dissolving the produced formazan crystals. Spectrophotometric measurements of the absorbance were made at wavelength of 570 nm, utilizing a microplate reader (Model 550, Bio-Rad, USA). The experiment was performed in triplicate. The IC_50_ values were then determined, and the results were evaluated in comparison with the standard drug doxorubicin (D1515, Sigma-Aldrich, Germany), used at concentration of 100 µg/mL.

### Computational study

#### Network pharmacology-based analysis

#####  Screening for the targets of UR1 and UR2 compounds

The target genes of compounds characterized in the extracts derived from *Aspergillus* sp. (UR1) and *Penicillium* sp. (UR2) were obtained through searching in the Traditional Chinese Medicine Systems Pharmacology Database, Analysis Platform (TCMSP) database (https://old.tcmsp-e.com/index.php)^35^ and Swiss Target Prediction Database (http://www.swisstargetprediction.ch/), with regard to chemical similarities, protein interactions and pharmacophore models. Afterwards, these target genes were transformed into their conical gene names with the use of UniProt database (https://www.uniprot.org/)^36^.

##### Screening of HepG2 and CaCo-2 cancer cell lines associated target genes

Genes linked to colorectal adenocarcinoma and hepatocellular carcinoma in human were acquired from GeneCards (www.genecards.org)^37^ as well as the National Center for Biotechnology Information (NCBI) (https://www.ncbi.nlm.nih.gov)^38^ adopting the keywords " human colorectal adenocarcinoma and CaCo-2, hepatocellular carcinoma and HepG2” and species limited to “Homo sapiens”. Following the elimination of duplicate targets, overlapping component-related and disease-related proteins were determined using Venny (https://bioinfogp.cnb.csic.es/tools/venny/)^39^ intersections to anticipate the overlap among the target genes impacted by UR1 and UR2 ingredients and the potential target genes associated with HepG2 and CaCo-2 cancer cell lines.

##### Constructing protein–protein interaction (PPI) network

A query list of target genes was used to create a PPI network with STRING version 12.0 (https://string-db.org/)^40^, which was then exported to the Cytoscape software version 3.10.1 (USA)^41^, a free software program designed to visualize, model and analyze the networks of molecular and genetic interactions (confidence score = 0.400). Cytohubba plug-in was used for screening of the top ten important genes.

####  Molecular docking

The crystal structures of the potential target genes were obtained using the Protein Data Bank, **EGFR** (PDB ID: 1M17), **PPARG** (PDB ID: 7AWD), **ESR1** (PDB ID: 7UJW), and **GSK3B** (PDB ID: 5K5N)^42^. AutoDockTools was utilized to create the input files for the dereplicated secondary metabolites, protein structures and cocrystallized ligands^43^, whereas OpenBable v2.4 was employed to process all ligands^44^. Molecular docking of the compounds to the proteins under investigation was conducted via AutoDock Vina^45^. Grid boxes that were not larger than 27,000 3 were chosen, and the “exhaustiveness” was set to 32, that is the recommended value for employing small boxes. For the purpose of validating the docking process, shown in S1 Fig, co-crystallized ligands were redocked for protein crystal structures in complexes with binding molecules. With the aid of Discovery Studio Visualizer 17.2.0, the interactions between docked compounds and the key proteins were examined.

## Results

### Isolation and identification of the marine sponge-associated fungi and phylogenetic analysis

The phylogenetic identification of the two strains depending on partial 18 S rRNA gene and ITS sequences showed that the strains represent an *Aspergillus* sp. (UR1) and a *Penicillium* sp. (UR2). Strain UR1 shared the highest partial 18 S rRNA gene sequence identity (99.39%) with *Aspergillus nidulans* ATCC 10074 (NG_064803) and the highest ITS sequence identity (99.80%) with *Aspergillus pseudodeflectus* NRRL 6135 (NR_135372.1). Strain UR2 shared highest partial 18 S rRNA gene sequence identity (99.8%) with *Penicillium limosum* CBS 339.97 (NG_062729.1) followed by strains of three other species with 99.7% (*Penicillium samsonianum*,* Penicillium subarcticum*, and *Penicillium tricolor*) and highest ITS sequence identity (99.81%) with *Penicillium oxalicum* NRRL 787 (NR_121232.1). The phylogenetic placement of the strains to the next related species was confirmed by the calculated phylogenetic trees shown in S2 and S3 Figures.

### Metabolomics profiling of the fungi *Aspergillus* sp. (UR1) and *Penicillium* sp. (UR2) associated with the marine sponge *Spheciospongia vagabunda*

**Fig. 1:**
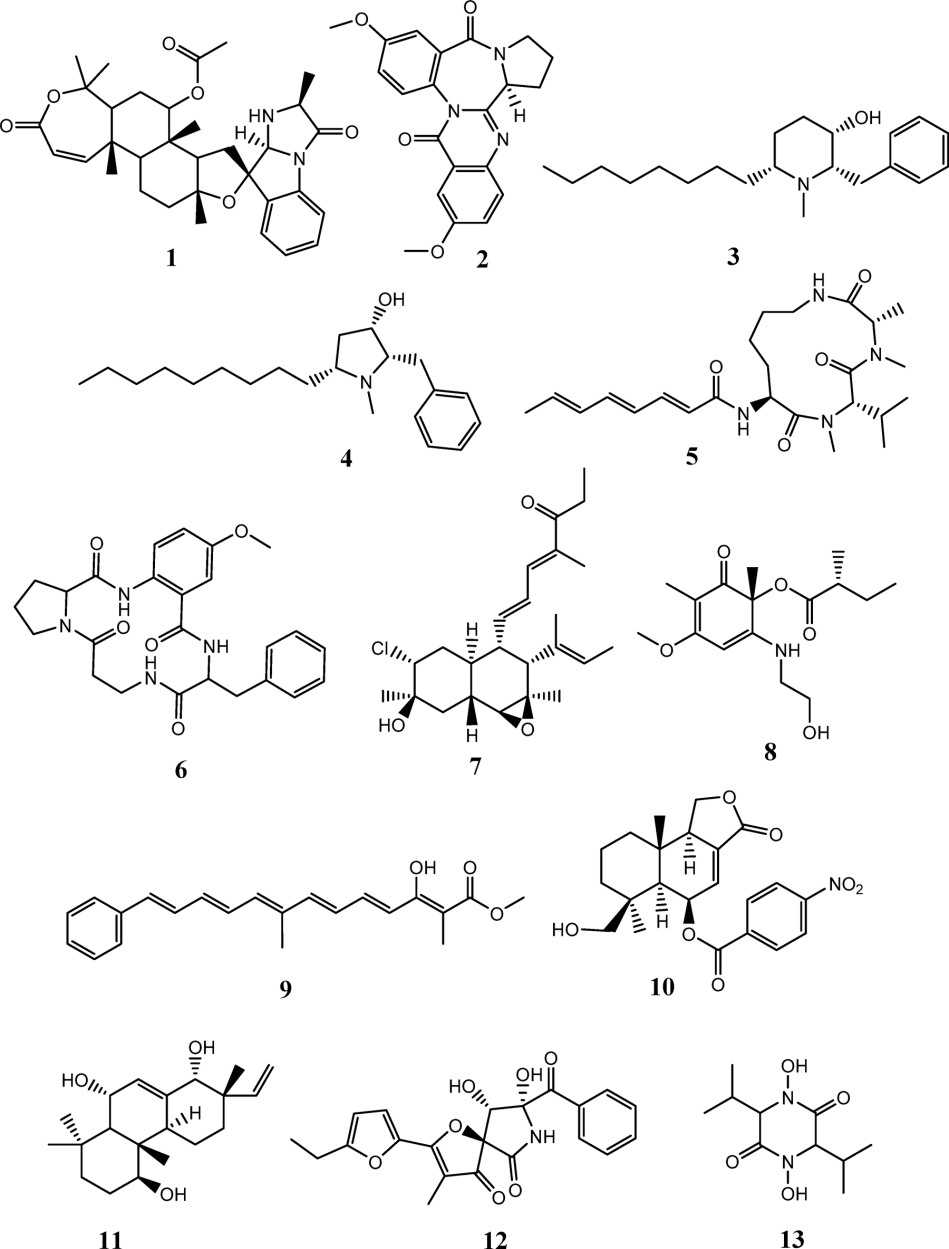
The dereplicated compounds (1-13) in the total extract of the Red Sea sponge-derived *Aspergillus* sp. (UR1).

**Fig. 2:**
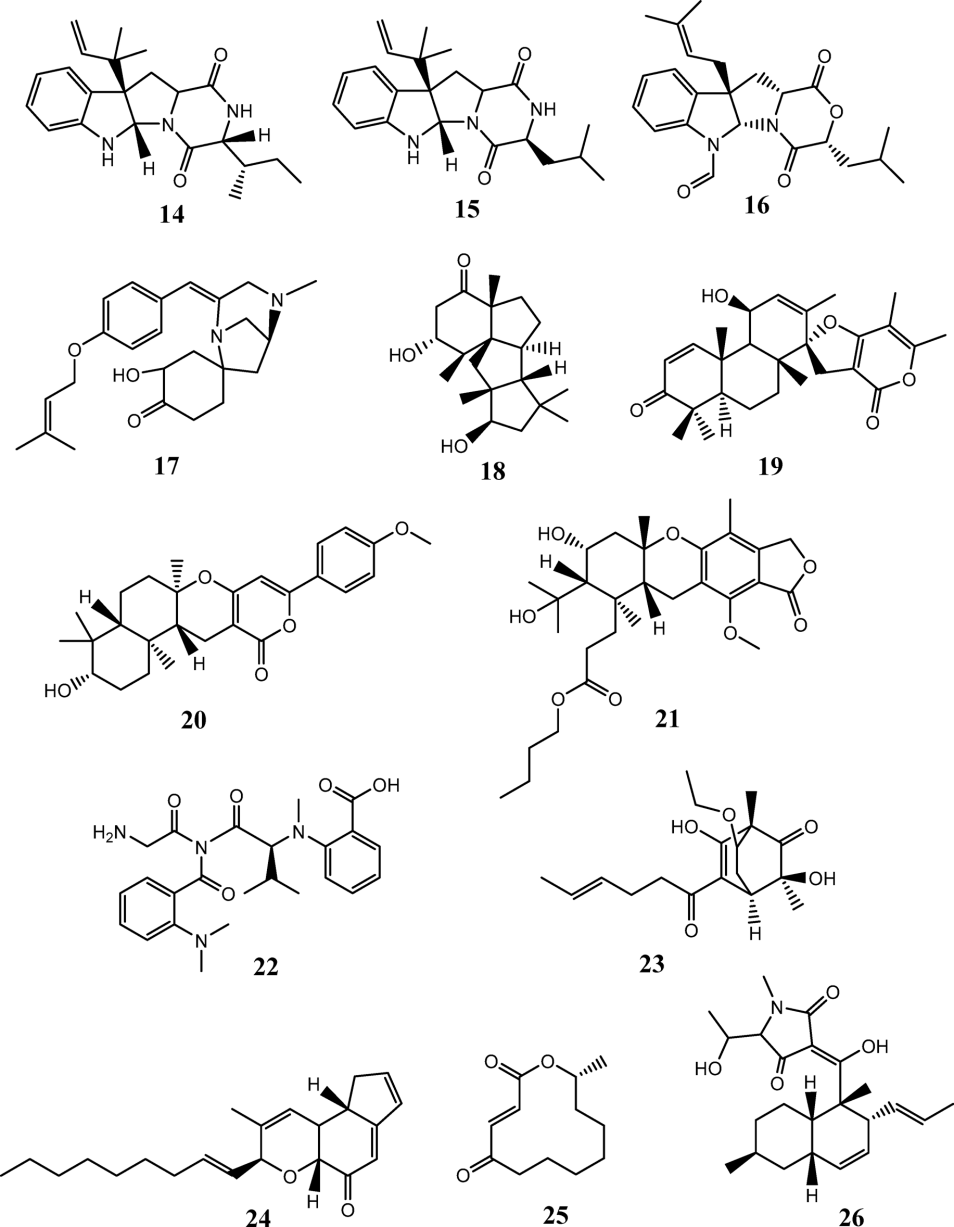
The dereplicated compounds (14-26) in the total extract of the Red Sea sponge-derived *Penicillium* sp. (UR2).

Metabolomic analysis is a very useful technique for chemical profiling and fingerprinting for complex crude extracts of natural origins. It is considered a large-scale analysis for secondary metabolites and serves primarily in the annotation and dereplication of major and minor constituents. Correspondingly, it plays a crucial part in the successful investigation of natural products chemo-diversity for drug discovery^46,47^. Considering this, the total extracts of the fungi *Aspergillus* sp. (UR1) and *Penicillium* sp. (UR2), purified from the sponge *Spheciospongia vagabunda*, were subjected to untargeted LC-HR-ESI-MS-based metabolomic profiling for the aim of dereplication. Both negative and positive modes of ionization were implemented to maximize the possible detection of secondary metabolites which could differ according to their ionization potential and physical characteristics (S4-S7 Figs). The tentative identification of secondary metabolites was accomplished through searching some databases namely DNP and Marinlit. S1 Table and Figs. 1 and 2 summarize the dereplicated metabolites of the marine-derived fungal strains obtained from the sponge *Spheciospongia vagabunda*.

#### Chemical dereplication of *Aspergillus* sp. (UR1)

Metabolomic analysis of *Aspergillus* sp. (UR1) total extract has brought about the putative identification of 13 diverse compounds, dereplicated mainly as alkaloids, cyclic peptides and polyketides. The mass ion peak at *m/z* 563.3117 [M + H]^+^ for the assumed molecular formula C_33_H_42_N_2_O_6_ was characterized as teraspiridole A (**1**), an alkaloid that had previously been purified from the creek-bottom-derived fungus *Aspergillus terreus*^48^. Another alkaloid, of benzodiazepine type, was identified as circumdatin J (**2**), according to the mass ion peak at *m/z* 376.1298 [M-H]^−^and the molecular formula C_21_H_19_N_3_O_4_. This compound had earlier been isolated from the marine fungus *Aspergillus ostianus*^49^. Whereas, the compound with the molecular formula C_21_H_35_NO was dereplicated as the piperidine alkaloid asperidine B (**3**), and/or the pyrrolidine alkaloid preussin (**4**) from the mass ion peak at 318.2800 [M + H] ^+^. The former has previously been obtained from the soil-derived *Aspergillus sclerotiorum* PSU-RSPG178^50^, and the latter has been purified earlier from the fungus *Simplicillium lanosoniveum* TAMA 173^51^. Likewise, a cyclic tripeptide was characterized as sclerotiotide C (**5**), concurring with the mass ion peak at *m/z* 447.2957 [M + H]^+^ and the molecular formula C_24_H_38_N_4_O_4_; it had earlier been purified from the salt sediment-derived fungus *Aspergillus sclerotium* PT06-1^52^. Whereas, the mass ion peak at *m/z* 465.2145 [M + H]^+^, corresponding to the suggested molecular formula C_25_H_28_N_4_O_5_, was characterized as aspercolorin (**6**), a cyclic tetrapeptide that had previously been obtained from *Aspergillus versicolor*^53,54^.

Alongside the previously stated compounds, two polyketides were dereplicated with mass ion peaks at *m/z* 407.2345 and 312.1804 [M + H]^+^, identified as ICM0301 C (**7**), previously obtained from *Aspergillus* sp. F-1491^55^, and wasabidienone E (**8**), formerly obtained from the sponge-derived *Aspergillus flocculosus* 01nt.1.1.5^56^, matched with the molecular formulas C_24_H_35_ClO_3_ and C_16_H_25_NO_5_, respectively. Moreover, the mass ion peak at *m/z* 337.1789 [M + H]^+^, concurring with the predicted molecular formula C_22_H_24_O_3_, was identified as asperrubrol (**9**), a phenylpolyene compound formerly obtained from *Aspergillus niger*^57^. The sesquiterpenoid nitrobenzyl ester with the molecular formula C_22_H_25_NO_7_ was characterized as 14-hydroxy-6β-*p*-nitrobenzoyl-cinnamolide (**10**) from the mass ion peak at *m/z* 414.1562 [M-H]^−^. It was previously purified from the marine green alga-derived *Aspergillus versicolor*^58^. Furthermore, metabolomics analysis of *Aspergillus* sp. (UR1) has brought about the characterization of a diterpenoid with norcleistanthane-type skeleton, aspergiloid D (isopimarane) (**11**) in consonance with the mass ion peak at *m/z* 321.2429 [M + H]^+^ and the molecular formula C_20_H_32_O_3_, which had previously been isolated from the endophyte *Aspergillus* sp. YXf3^59^. Azaspirofuran B (**12**), a hetero-spirocyclic γ-lactam previously reported from the marine sediment-derived *Aspergillus sydowii* D2-6, was also dereplicated from the mass ion peak at *m/z* 396.1078 [M-H]^−^ coinciding with the predicted formula C_21_H_19_NO_7_^60,61^. Further, the mass ion peak at *m/z* 231.1346 [M + H]^+^, matched to the formula C_10_H_18_N_2_O_4_, was characterized as terramide C (**13**), a piperazine-2,5-dione, where hydroxamic acid residues make up both of the amino acid components. It was previously obtained from *Aspergillus terreus* CMI 44,339^62^.

#### Chemical dereplication of *Penicillium* sp. (UR2)

Chemical profiling of the marine-derived fungus *Penicillium* sp. (UR2) total extract has led to the detection of 13 compounds, demonstrating that this fungus is a prolific reservoir of different metabolites such as diterpenoids, meroterpenoids and alkaloids. The alkaloid having the molecular formula C_22_H_29_N_3_O_2_ was dereplicated as brevicompanine A (**14**) and/or *allo*-brevicompanine B (**15**) from the mass ion peak at *m/z* 368.2323 [M + H]^+^. These are diketopiperazine alkaloids previously obtained from *Penicillium brevicompactum*^63^ and the deep-ocean sediment-derived *Penicillium* sp.^64^, respectively. Another closely related compound designated as mollenine B (**16**), was identified on the basis of the mass ion peak at *m/z* 397.2118 [M + H]^+^ and in consonance with the molecular formula C_23_H_28_N_2_O_4_. This dioxomorpholine-type alkaloid had been formerly found in *Eupenicillium molle* NRRL 13,062^65^. One more alkaloid, tyrosine-derived, was identified as TAN-1251B (**17**) considering the mass ion peak at *m/z* 397.2476 [M + H]^+^and the molecular formula C_24_H_32_N_2_O_3_, it had previously been discovered in *Penicillium thomii* RA-89^66^.

Besides the aforementioned compounds, metabolomic analysis of *Penicillium* sp. revealed the presence of diterpenoids. As an example, a cyclopiane-class diterpene was dereplicated as conidiogenone H (**18**) in light of the determined mass ion peak at *m/z* 321.2429 [M + H]^+^and according to the molecular formula C_20_H_32_O_3_. This compound had formerly been reported from the marine red alga-derived *Penicillium chrysogenum* QEN-24 S^67^. Another spiro-diterpenoid was identified as brevione I (**19**), in response to the determined mass ion peak at *m/z* 439.2483 [M + H]^+^, and in compliance with the molecular formula C_27_H_34_O_5_. This diterpenoid had been earlier purified from the deep-sea sediment-derived *Penicillium* sp^68^. Moreover, a meroterpenoid, arisugacin F (**20**), in agreement with the mass ion peak at *m/z* 439.2483 [M + H]^+^ and the molecular formula C_27_H_34_O_5_, which had previously been isolated from the endophytic fungus *Penicillium* sp. SXH-65, was also detected^69^. Whereas the mass ion peak at *m/z* 519.2962 [M + H]^+^, complying with the assumed molecular formula C_29_H_42_O_8_, was dereplicated as austalide H acid butyl ester (**21**), another meroterpenoid formerly obtained from the marine brown alga-derived *Penicillium thomii* KMM 4645^70^. Also, the mass ion peak at *m/z* 455.2285 [M + H]^+^ for the anticipated molecular formula C_24_H_30_N_4_O_5_ was identified as the tetrapeptide viridic acid (**22**), a mycotoxin that had been isolated before from *Penicillium viridicatum*^71^. Meanwhile, the mass ion peak at *m/z* 323.1845 [M + H]^+^, agreeing with the estimated molecular formula C_18_H_26_O_5_, was dereplicated as the polyketide rezishanone D (**23**), earlier obtained from *Penicillium notatum*^72^. Furthermore, a benzopyran derivative that had formerly been reported from the marine green alga-derived *Penicillium sp.*^73,74^ was dereplicated as penostatin C (**24**), matched with the mass ion peak at *m/z* 325.2179 [M-H]^−^ and the molecular formula C_22_H_30_O_2_. Additionally, one 12-membered macrolide was identified according to the mass ion peak at *m/z* 211.1323 [M + H]^+^, affirming the formula C_12_H_18_O_3_, as patulolide A (**25**), it had already been isolated from *Penicillium urticae* SllR59^75^. Finally, a methylpyrrolidine-2,4-dione derivative, characterized as cissetin (**26**), earlier purified from the endophytic fungus *Preussia* sp^76^. , was characterized from the mass ion peak at *m/z* 386.2335 [M-H]^−^, in consonance with the formula C_23_H_33_NO_4_.

###  Cytotoxic activity

The crude extracts of the two fungi *Aspergillus* sp. (UR1) and *Penicillium* sp. (UR2), purified from the Red Sea sponge *S. vagabunda*, were in vitro assessed for their cytotoxic activity implementing MTT reduction colorimetric assay. Three different cancer cell lines; hepatocellular carcinoma (HepG2), human breast cancer (MCF7) and human colon carcinoma (CaCo-2) were used in this evaluation. The *Aspergillus* sp. (UR1) extract showed the highest antiproliferative activity with IC_50_ values of 2.61 ± 0.12, 3.23 ± 0.21, and 3.41 ± 0.18 µg/ml against HepG2, CaCo-2 and MCF7, respectively. Whereas *Penicillium* sp. (UR2) extract exhibited a less potent effect with IC_50_ values 17.65 ± 0.28, 18.38 ± 0.19, and 22.45 ± 0.27 µg/ml, against the same cancerous cell lines. All results are included in Table 1.

**Table 1:** Cytotoxic potential of the marine spong-derived fungi *Aspergillus* sp. (UR1) and *Penicillium* sp. (UR2) total extracts against different cancer cell lines.

Code	IC_50_ (µg/ml) HepG2	IC_50_ (µg/ml) MCF7	IC_50_ (µg/ml) CaCo-2
*Aspergillus* sp. UR1	2.61 ± 0.12	3.41 ± 0.18	3.23 ± 0.21
*Penicillium* sp. UR2	17.65 ± 0.28	22.45 ± 0.27	18.38 ± 0.19
Doxorubicin	1.32 ± 0.06	1.72 ± 0.03	2.12 ± 0.04

### Computational study

#### Network pharmacology-based analysis

The significant cytotoxic activity observed, particularly targeting hepatocellular carcinoma (HepG2) and human colon carcinoma (CaCo-2), in the extracts derived from *Aspergillus* sp. (UR1) and *Penicillium* sp. (UR2) underscore the potential of network pharmacology. This approach proves efficacious in constructing a comprehensive network linking compounds, proteins/genes, and diseases, all in a high-throughput fashion^77,78^.

##### Screening for the targets of UR1 and UR2 compounds

The target genes of UR1 and UR2 ingredients were recuperated from the Swiss Target Prediction and TCMSP databases, including 473 genes related to UR1 ingredients and 432 genes related to UR2 ingredients. The UniProt database was then used to convert these genes into their canonical gene names.

##### Screening of HepG2 and CaCo-2 cancer cell lines associated target genes

**Fig. 3:**
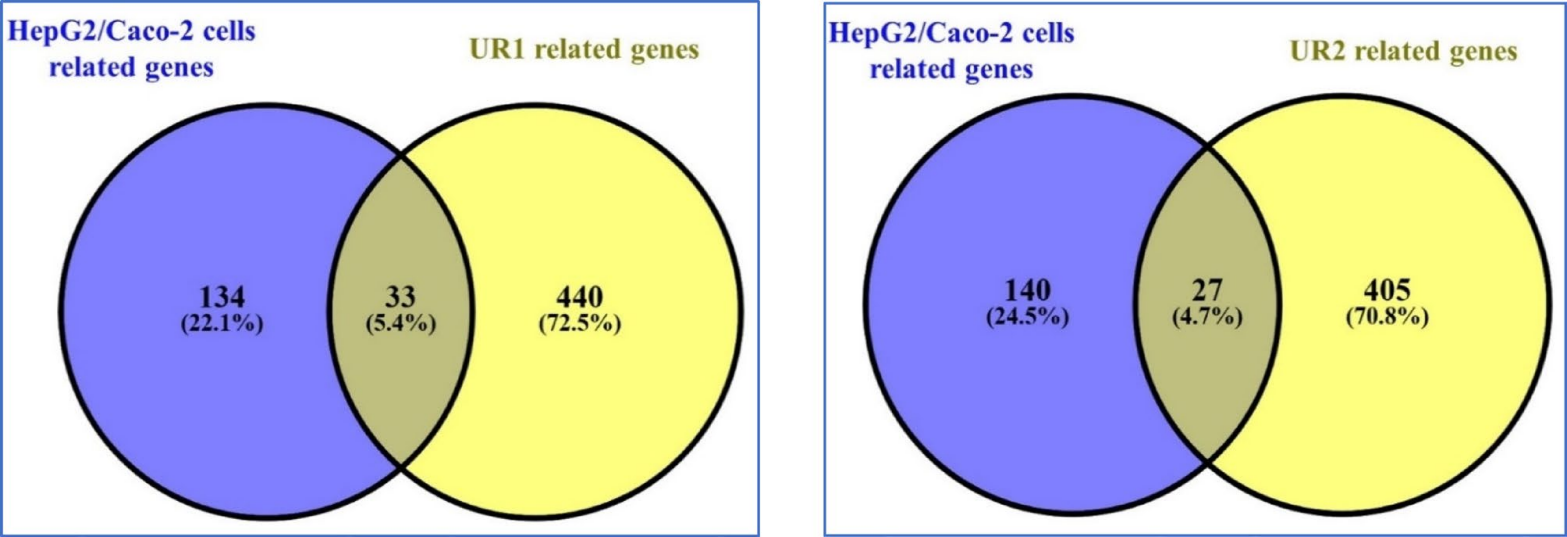
Venn diagram for the integrated analysis of the associated targets of UR1 and UR2 ingredients and HepG2/Caco-2 cancer cell-related genes.

The most common **167** target genes after removing duplicates are included in both HepG2 and CaCo-2 cancer cell lines and were retrieved from the databases GeneCards and NCBI. Particular search criteria were implemented to extract these genes, including the keywords “human colorectal adenocarcinoma and **CaCo-2**, hepatocellular carcinoma and **HepG2**” with a species restriction limited to “Homo sapiens”. The overlap between the target genes impacted by UR1 and UR2 components and the potential target genes associated with HepG2 and CaCo-2 cancer cell lines, was carefully visualized through creating a Venn diagram. The visual representation of two networks shared targets, as illustrated in Fig. 3. After eliminating duplicates, a set of **20** vital gene targets that UR1 and UR2 ingredients have in common and effectively target HepG2 and CaCo-2 cancer cell lines were identified as the most prominent targets.

##### Constructing protein–protein interaction (PPI) network

**Fig. 4:**
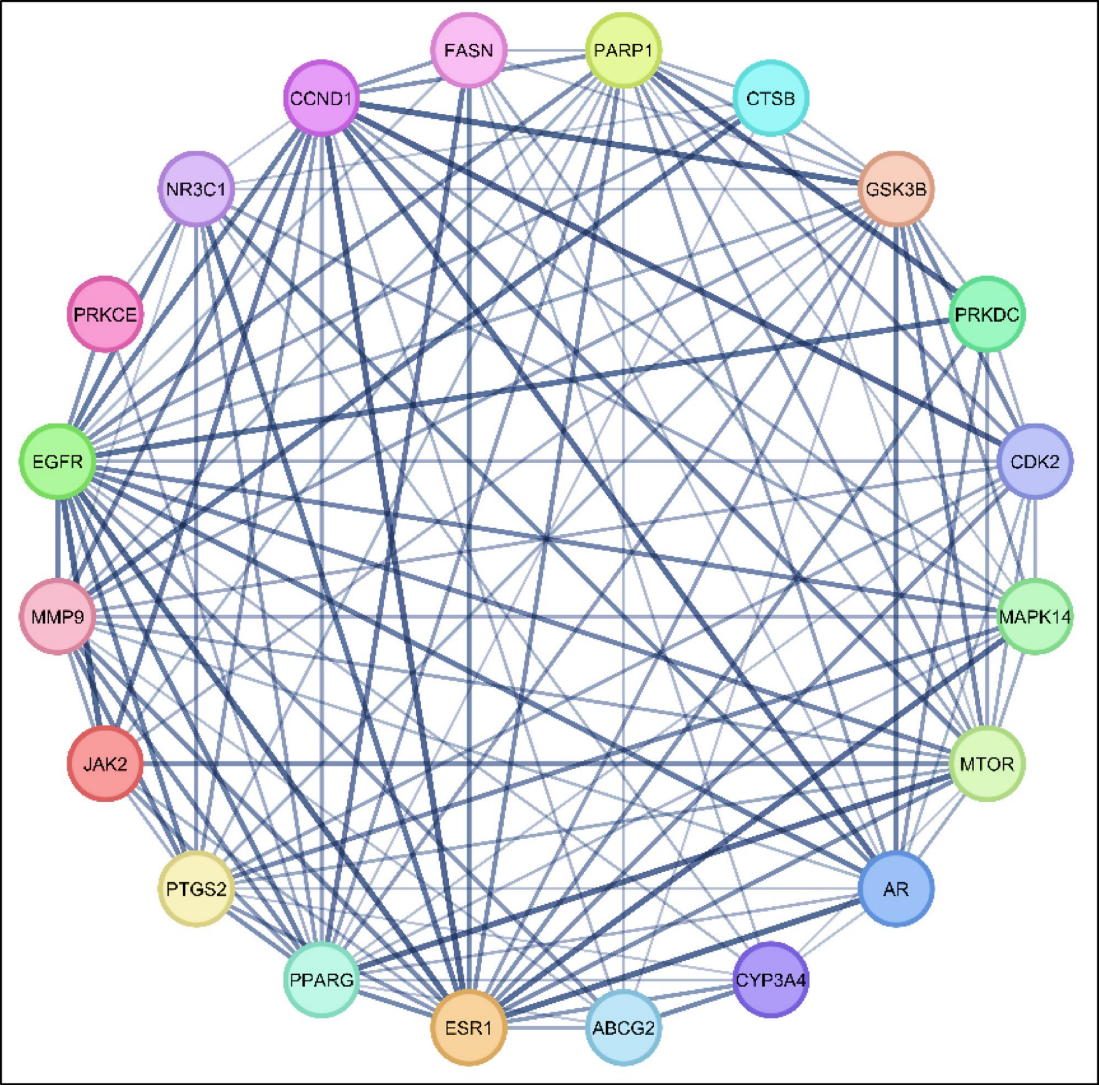
Twenty protein targets represented by network nodes, and protein–protein interactions represented by the edges.

**Fig. 5:**
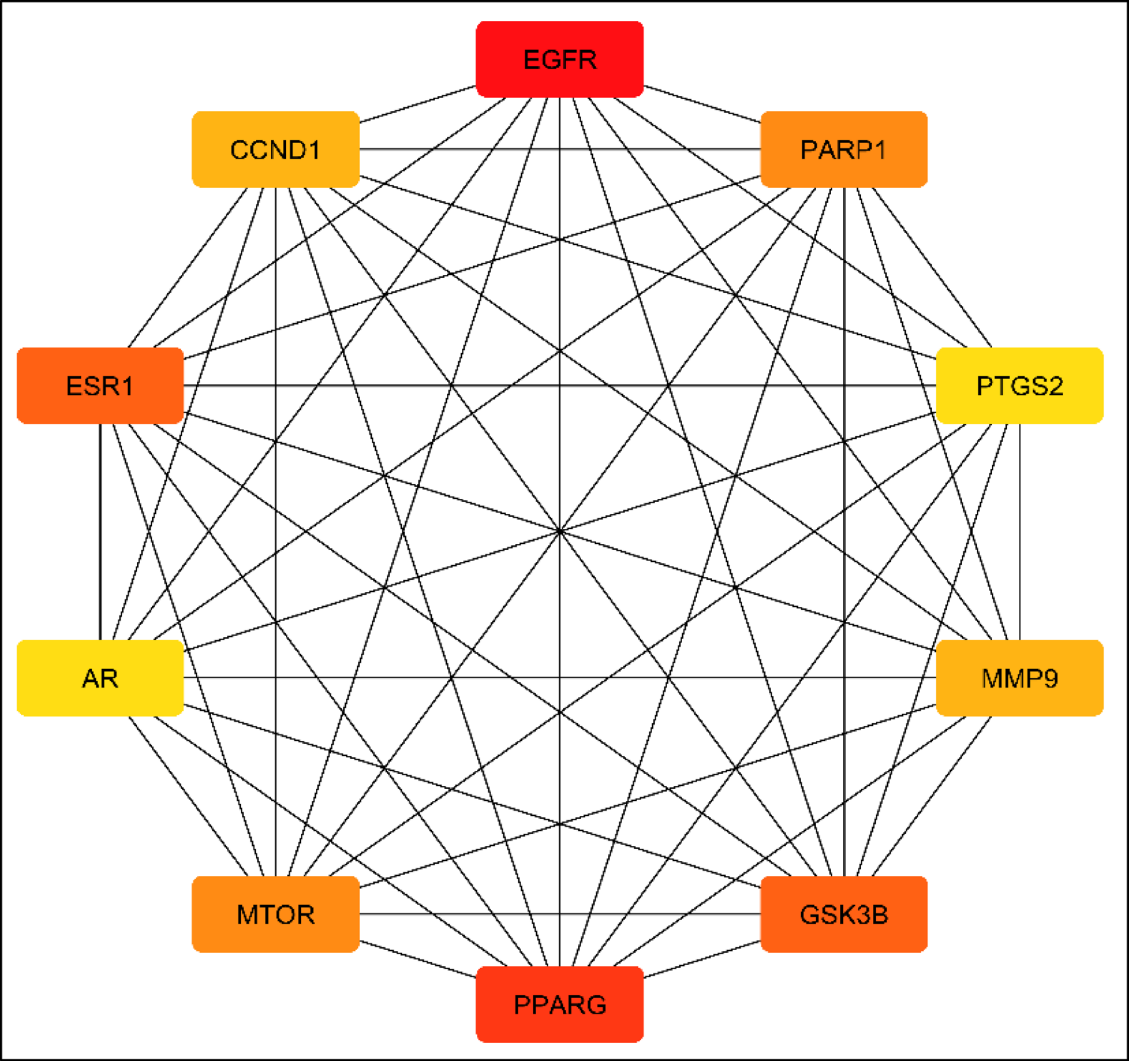
The top ten hub genes represented by network nodes: higher score and stronger connections are associated with darker colors..

A protein-protein interaction (PPI) network was built up by submitting the 20 commonly identified targeted genes to the STRING database. The obtained results were utilized to produce a graphical representation of the PPI network *via* the use of Cytoscape 3.10.1 software. This network comprised 20 nodes and 124 edges, the average node connectivity was 12.40, as demonstrated in Fig. 4. The top ten important genes were then identified and extracted employing the Cytohubba plugin according to their connectivity degree throughout the network, as illustrated in Fig. 5. The higher the degree value of any gene, the more apparent it will be in the pathogenesis of the disease. These genes, in ascending manner, are EGFR, PPARG, ESR1, GSK3B, MTOR, PARP1, MMP9, CCND1, AR and PTGS2. S2 Table provides a summary of the topological parameters, comprising node degree, closeness and betweenness and for each protein.

#### Molecular docking

Docking studies were performed to investigate the binding between potential targets and active compounds that were detected within the UR1 and UR2 extracts. The identified compounds were undergoing molecular docking within the active sites of the top four hub genes, namely **EGFR**, **PPARG**, **ESR1**, and **GSK3B** were selected to represent the four different pathways. Firstly, we validated the docking protocol through performing re-docking of co-crystallized ligands, establishing that the RMSD was less than 2 Å, between the crystal pose and the docked pose, for a successful validation S1 Fig. Then, we docked the extracted compounds into these hub genes and the obtained docking scores are presented in S3 Table.

The docking analysis results indicated that the identified compounds in both extracts of *Aspergillus* sp. (UR1) and *Penicillium* sp. (UR2) exhibit favorable binding affinity scores with selected hub genes in comparison to the co-crystallized ligands associated with these genes. In particular, the binding modes of complexes involving EGFR-compound **13** (-6.27 kcal/mol), PPARG-compound **6** (− 9.13 kcal/mol), ESR1-compound **7** (− 7.86 kcal/mol), and GSK3B-compound **2** (− 5.95 kcal/mol) correspond to extracts from UR1. On other hands, UR2 extracts show variable binding affinity within these active sites especially, EGFR-compound **21** (− 6.23 kcal/mol), PPARG-compound **16** (-8.38 kcal/mol), ESR1-compound **15** (− 8.21 kcal/mol), and GSK3B-compound **25** (− 6.76 kcal/mol).

**Fig. 6:**
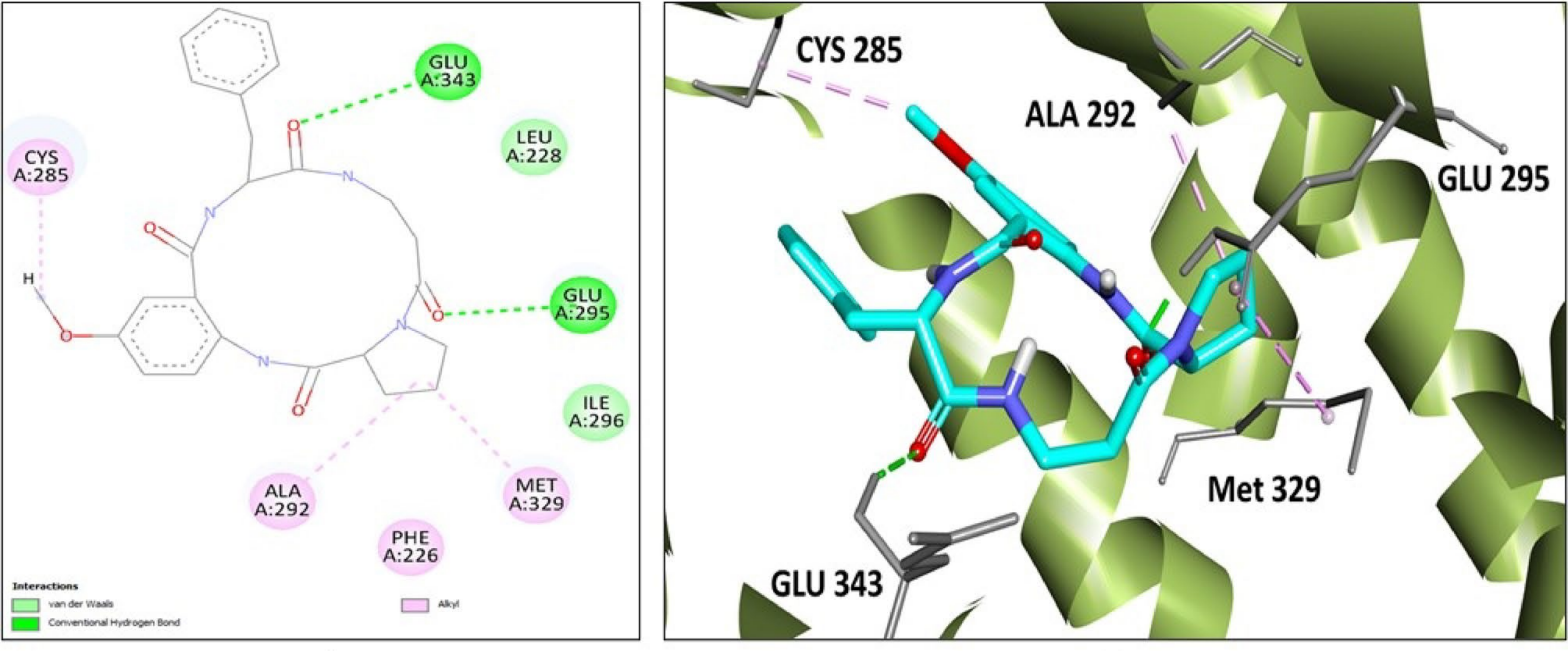
2D and 3D docking interaction diagrams of compound 6 in PPARG active site (PBD ID 7AWD).

It was observed from docking results that all compounds extracted from both UR1 and UR2 exhibited strong to outstanding binding affinity with the PPARG gene when compared to other hub genes. For instance, compound **6** (identified in UR1 extract) binds to PPARG (with S value= − 9.13 kcal/mol) through forming two hydrogen bond interactions with Glu 295 and Glu 343 amino acid residues as well as forming three hydrophobic interactions with Cys 285, Ala 292 and Met 329 amino acid residues Fig. 6.

**Fig. 7:**
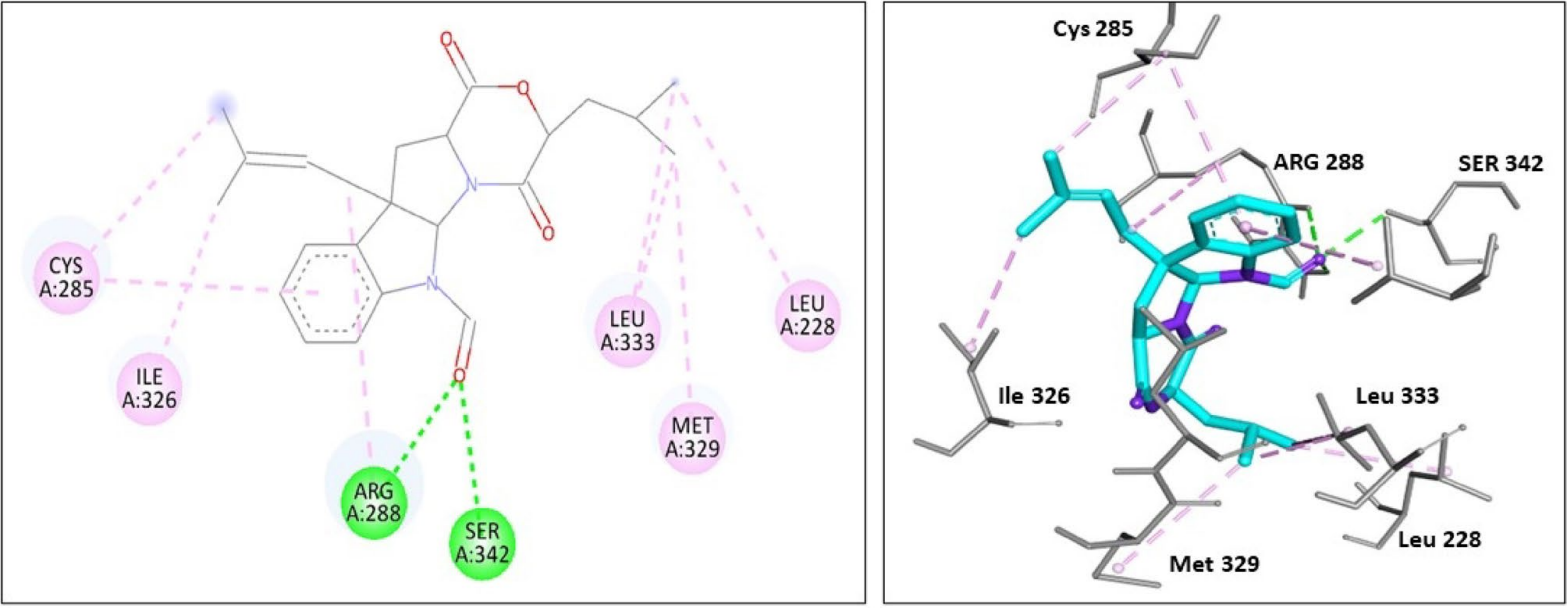
2D and 3D docking interaction diagrams of compound 16 in PPARG active site (PBD ID 7AWD).

In addition, compound **16** (identified in UR2 extract) binds to PPARG (with S value= − 8.38 kcal/mol) via forming three hydrogen bond interactions with Arg 288 and Ser 342 amino acid residues. Also, it showed five hydrophobic interactions with Leu 228, Cys 285, Ile 326, Met 329, and Leu 333 amino acid residues (Fig. 7).

## Discussion

Sponge-associated fungi have been shown to be abundant sources of metabolites with distinctive structures and biological activities. We investigate, herein, the chemical profiles of the total extracts of two fungal strains purified from the Red Sea-derived sponge *S. vagabunda*.

The fungal strains were isolated from the sponge and were identified, according to the 18 S rRNA gene sequence analysis, as *Aspergillus* sp. (UR1) and *Penicillium* sp. (UR2). The strains were then cultured, and each culture was extracted with ethyl acetate. The resulting extracts were submitted to metabolomics evaluation through LC-HR-ESI-MS. Chemical profiling of *Aspergillus* sp. (UR1) extract has resulted in the characterization of 13 metabolites, dereplicated mainly as alkaloids, cyclic peptides and polyketides. Whereas, the chemical profiling of *Penicilluim* sp. (UR2) extract brought about the putative identification of 13 compounds of various classes comprising alkaloids, diterpenoids and meroterpenoids.

Furthermore, the cytotoxic activity of both extracts was assessed, applying the MTT colorimetric reduction assay, against 3 cancer cell lines; HepG2, CaCo-2 and MCF7. *Aspergillus* sp. (UR1) extract displayed higher antiproliferative activity with IC_50_ values 2.61 ± 0.12, 3.23 ± 0.21 and 3.41 ± 0.18 µg/ml against HepG2, CaCo-2 and MCF7, respectively. Similarly, *Penicillium* sp. (UR2) extract revealed a less potent effect with IC_50_ values of 17.65 ± 0.28, 18.38 ± 0.19, and 22.45 ± 0.27 µg/ml, against the same cancerous cell lines. The presence of cytotoxic secondary metabolites in the investigated crude extracts might provide an explanation for the observed cytotoxic potential. Apparently, many of the identified compounds have been reported in earlier studies to display cytotoxicity towards different cancer cell lines, which may eventually support the conclusion of our investigation. For instance, compound **4** was found to exhibit significant cytotoxicity against the triple-negative breast cancer cell line (MDA-MB231)^79^. Whereas, compound **7** has been shown to display weak cytotoxic effect towards Hela S3 and NCI-H69 cells^80^. In addition, compound **10** has been reported to exert significant cytotoxicity against HCT-116 human colon carcinoma cells and moderate selective toxicity towards a panel of renal tumour cell lines^58^, and compound **12** was proved to exhibit notable cytotoxic and antitumour activities^81^. Furthermore, it has been reported that compound **15** showed weak inhibitory effect on five human cancer cell lines (K-562, HL-60, Hela, BGC-823 and MCF-7)^82^. However, compound **19** has been documented to display significant cytotoxic activity against MCF-7 comparable to the positive control cisplatin^68^. Moreover, previous studies also reported that compound **20** demonstrated weak cytotoxicity towards Hela, HL-60 and K-562 cell lines^69^, and compound **24** has been also evidenced to exert significant cytotoxic effect against a panel of cancer cell lines^73^.

Additionally, in silico screening was carried out to fully comprehend the cytotoxic potential of the dereplicated metabolites in the fungal extracts. The compounds were undergoing molecular docking within the active sites of EGFR, PPARG, ESR1, and GSK3B genes (being the top four hub genes), representing four different pathways. The results demonstrated that most of the identified secondary metabolites have established strong binding affinities with the PPARG (Peroxisome proliferator-activated receptor gamma) gene. PPARG is one of the transcription factors that belongs to the nuclear receptor family. Its anti-proliferative and pro-apoptotic characteristics have been extensively documented across various cancers such as colon, esophageal, breast, lung, and prostate cancer^83^. Recent studies have demonstrated that activating endogenous or ectopically expressed PPAR-γ ligands is adequate to trigger growth arrest and apoptosis in diverse cancer cell lines^84^. Noteworthy, compound **6** (identified in UR1 extract) showed a binding energy to PPARG with S value of -9.13 kcal/mol, whereas compound **16** (identified in UR2 extract) showed a binding energy to PPARG with S value of -8.38 kcal/mol.

In summary, our study emphasized the prominence of the Red Sea sponge *S. vagabunda-*associated fungi as a valuable source of cytotoxic metabolites, which could promote future drug development for cancer management. Thus, additional studies are recommended.

## Electronic supplementary material

Below is the link to the electronic supplementary material.

Supplementary Material 1.

## Data Availability

The gene sequences (of strains UR1 and UR2) generated/analysed during the current study are available in the GenBank/EMBL/DDBJ with accession numbers PP843230-PP843231 (18 S rRNA gene sequences) and PP843228-PP843229 (ITS sequences).
