# Supplementary material for: Cytotoxicity evaluation and metabolomic profiling of Spheciospongia vagabunda-associated fungi corroborated by in silico studies
Source: Sci Rep. 2025 Jun 20;15:20115. doi: 10.1038/s41598-025-04162-6 (PMC12181285; doi:10.1038/s41598-025-04162-6)
Supplement: Supplementary file 1 — Supplementary Material 1. [file 41598_2025_4162_MOESM1_ESM.pdf]

Supporting Information

Cytotoxicity Evaluation and Metabolomic Profiling of *Spheciospongia vagabunda*-associated fungi Corroborated by *in silico* Studies

Omnia Hesham Abdelhafez[1¶], Miada F. Abdelwahab [2¶*], Abeer H. Elmaidomy [3], Arwa Mortada Ahmed[1], Mohamed Hisham[4], Stefanie P. Glaeser[5], Peter Kämpfer [5], Jun Wu[6], Usama Ramadan Abdelmohsen [1,2*]

[1] Department of Pharmacognosy, Faculty of Pharmacy, Deraya University, 61111 New Minia, Egypt

[2] Department of Pharmacognosy, Faculty of Pharmacy, Minia University, Minia 61519, Egypt

[3] Department of Pharmacognosy, Faculty of Pharmacy, Beni-Suef University, 62514 Beni-Suef, Egypt

[4] Department of Pharmaceutical Chemistry, Faculty of Pharmacy, Deraya University, 61111 New Minia, Egypt

[5] Institute of Applied Microbiology, Justus-Liebig University Giessen, D-35392 Giessen, Germany

[6] Guangdong Key Laboratory for Research and Development of Natural Drugs, College of Pharmacy, Guangdong Medical University, Dongguan 523808, China

* Corresponding authors

E-mail: usama.ramadan@mu.edu.eg (U.R.A.), mayada.mohamed2@mu.edu.eg (M.F.A)

[¶] These authors contributed equally to this work.

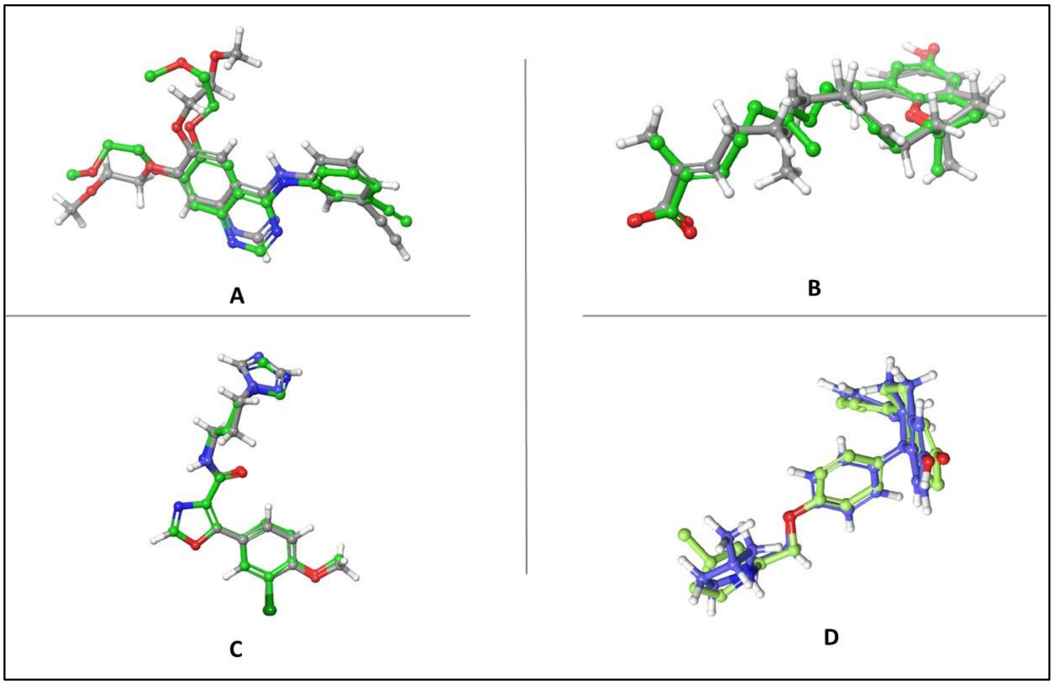

Figure S1. 3D view of redocked co-crystallized ligand for (A) **EGFR**, (B) **PPARG**, (C) **GSK3B**, (D) **ESR1**

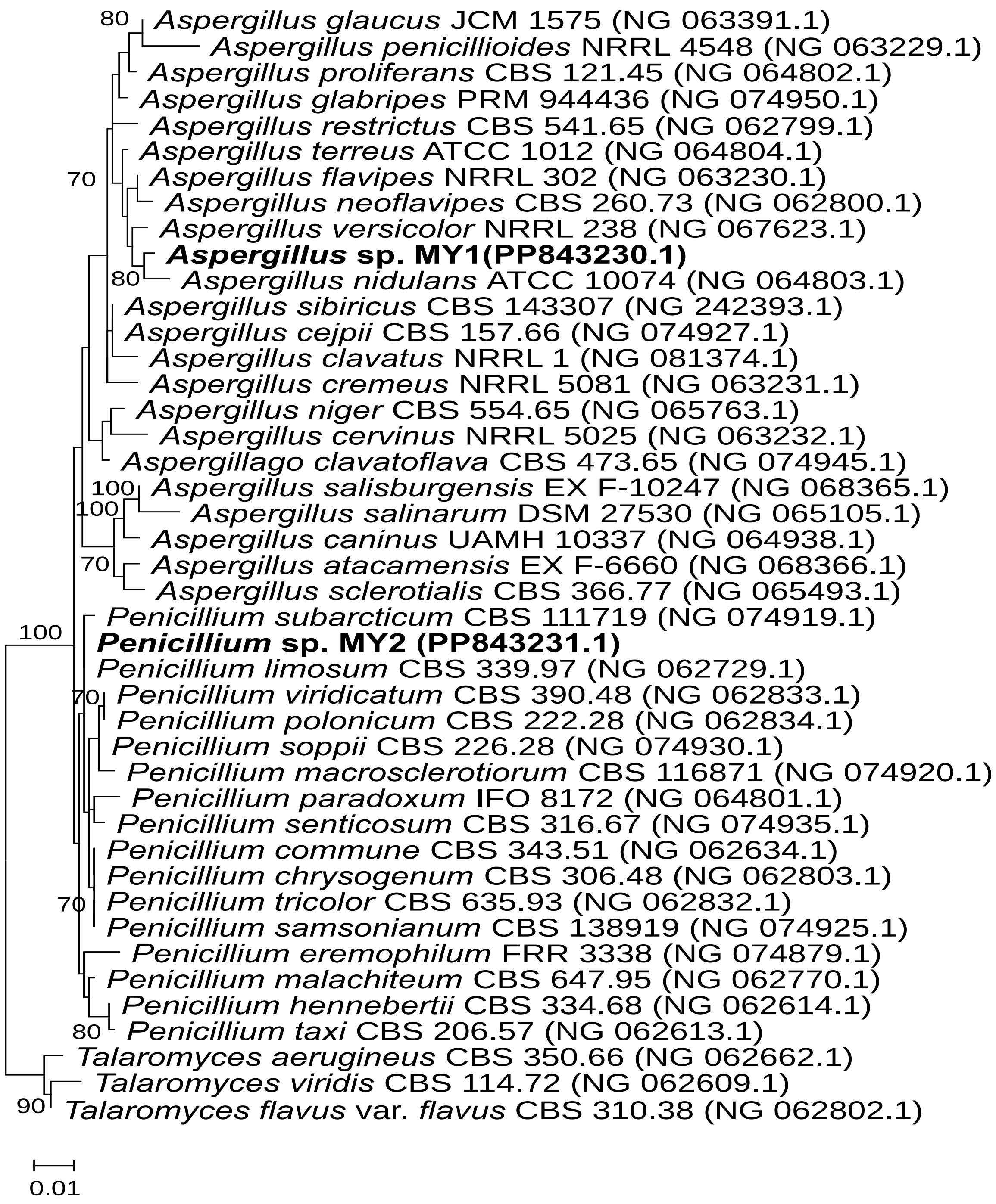

Figure S2. Phylogenetic placement of fungal strains UR1 and UR2 among next relates type strain material based on partial 18S rRNA gene sequences. The depicted tree represents a maximum likelihood tree which was calculated in MEGA11 considering 949 nucleotide positions. Numbers at nodes represent bootstrap values of 70% and above (based on 100 replicate trees). Numbers in brackets are Acc numbers of respective sequence. Bar: Substitution per nucleotide position.

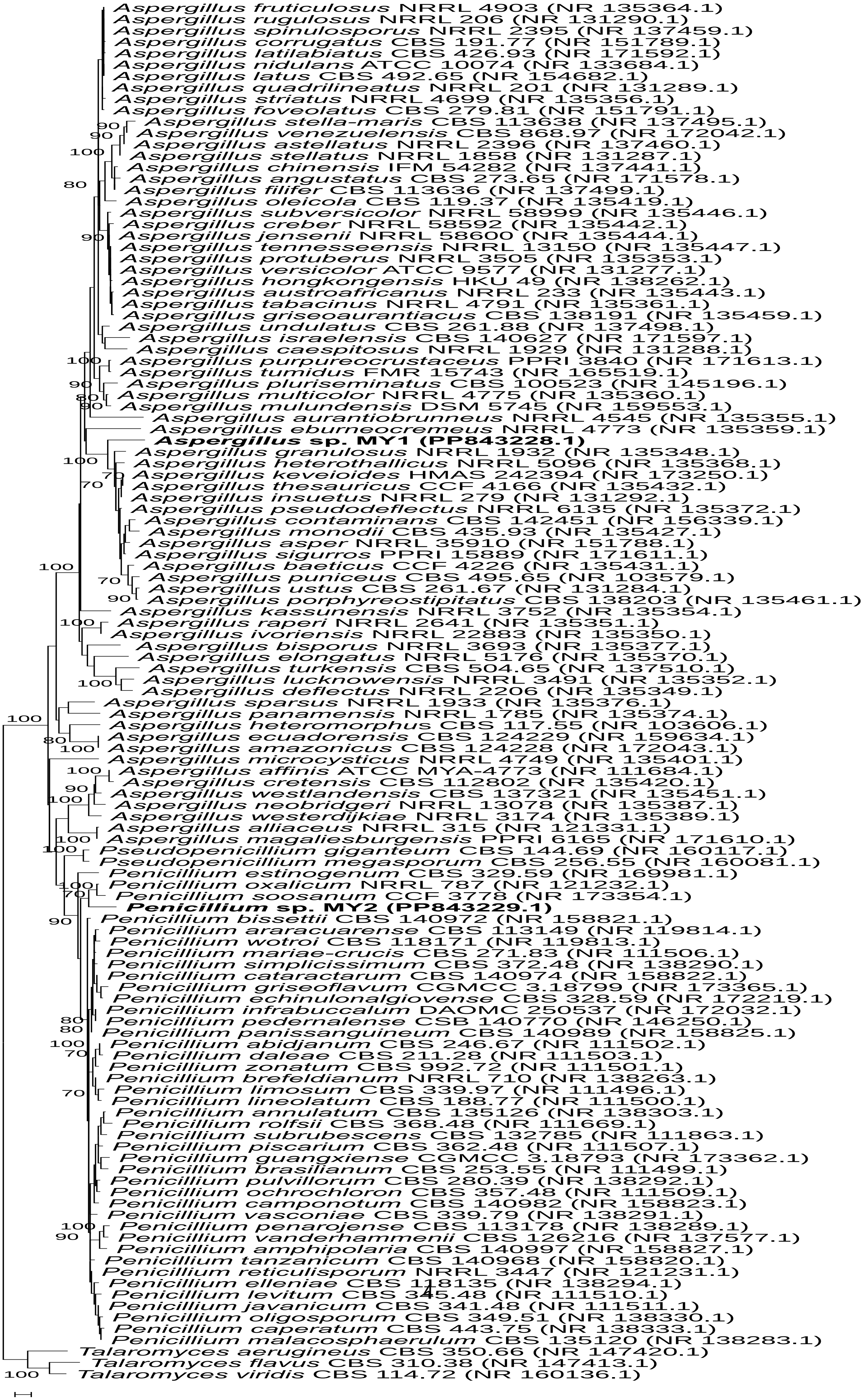
Aspergillus fruticulosus NRRL 4903 (NR 135364.1)
Aspergillus rugulosus NRRL 206 (NR 131290.1)
Aspergillus spinulosporus NRRL 2395 (NR 137459.1)
Aspergillus corrugatus CBS 191.77 (NR 151789.1)
Aspergillus latilabiatus CBS 426.93 (NR 171592.1)
Aspergillus nidulans ATCC 10074 (NR 133684.1)
Aspergillus latus CBS 492.65 (NR 154682.1)
Aspergillus quadrilineatus NRRL 201 (NR 131289.1)
Aspergillus striatus NRRL 4699 (NR 135356.1)
Aspergillus foveolatus CBS 279.81 (NR 151791.1)
Aspergillus stella-maris CBS 113638 (NR 137495.1)
Aspergillus venezuelensis CBS 868.97 (NR 172042.1)
Aspergillus astellatus NRRL 2396 (NR 137460.1)
Aspergillus stellatus NRRL 1858 (NR 131287.1)
Aspergillus chinensis IFM 54282 (NR 137441.1)
Aspergillus angustatus CBS 273.65 (NR 171578.1)
Aspergillus filifer CBS 113636 (NR 137499.1)
Aspergillus oleicola CBS 119.37 (NR 135419.1)
Aspergillus subversicolor NRRL 58999 (NR 135446.1)
Aspergillus creber NRRL 58592 (NR 135442.1)
Aspergillus jensenii NRRL 58600 (NR 135444.1)
Aspergillus tennesseensis NRRL 13150 (NR 135447.1)
Aspergillus protuberus NRRL 3505 (NR 135353.1)
Aspergillus versicolor ATCC 9577 (NR 131277.1)
Aspergillus hongkongensis HKU 49 (NR 138262.1)
Aspergillus austroafricanus NRRL 233 (NR 135443.1)
Aspergillus tabacinus NRRL 4791 (NR 135361.1)
Aspergillus griseoaurantiacus CBS 138191 (NR 135459.1)
Aspergillus undulatus CBS 261.88 (NR 137498.1)
Aspergillus israelensis CBS 140627 (NR 171597.1)
Aspergillus caespitosus NRRL 1929 (NR 131288.1)
Aspergillus purpureocrustaceus PPRI 3840 (NR 171613.1)
Aspergillus tumidus FMR 15743 (NR 165519.1)
Aspergillus pluriseminatus CBS 100523 (NR 145196.1)
Aspergillus multicolor NRRL 4775 (NR 135360.1)
Aspergillus mulundensis DSM 5745 (NR 159553.1)
Aspergillus aurantiobrunneus NRRL 4545 (NR 135355.1)
Aspergillus eburneocremeus NRRL 4773 (NR 135359.1)
Aspergillus sp. MY1 (PP843228.1)
Aspergillus granulosus NRRL 1932 (NR 135348.1)
Aspergillus heterothallicus NRRL 5096 (NR 135368.1)
Aspergillus keveioides HMAS 242394 (NR 173250.1)
Aspergillus thesauricus CCF 4166 (NR 135432.1)
Aspergillus insuetus NRRL 279 (NR 131292.1)
Aspergillus pseudodeflectus NRRL 6135 (NR 135372.1)
Aspergillus contaminans CBS 142451 (NR 156339.1)
Aspergillus monodii CBS 435.93 (NR 135427.1)
Aspergillus asper NRRL 35910 (NR 151788.1)
Aspergillus sigurros PPRI 15889 (NR 171611.1)
Aspergillus baeticus CCF 4226 (NR 135431.1)
Aspergillus puniceus CBS 495.65 (NR 103579.1)
Aspergillus ustus CBS 261.67 (NR 131284.1)
Aspergillus porphyreostipitatus CBS 138203 (NR 135461.1)
Aspergillus kassunensis NRRL 3752 (NR 135354.1)
Aspergillus raperi NRRL 2641 (NR 135351.1)
Aspergillus ivoriensis NRRL 22883 (NR 135350.1)
Aspergillus bisporus NRRL 3693 (NR 135377.1)
Aspergillus elongatus NRRL 5176 (NR 135370.1)
Aspergillus turkensis CBS 504.65 (NR 137510.1)
Aspergillus lucknowensis NRRL 3491 (NR 135352.1)
Aspergillus deflectus NRRL 2206 (NR 135349.1)
Aspergillus sparsus NRRL 1933 (NR 135376.1)
Aspergillus panamensis NRRL 1785 (NR 135374.1)
Aspergillus heteromorphus CBS 117.55 (NR 103606.1)
Aspergillus ecuadorensis CBS 124229 (NR 159634.1)
Aspergillus amazonicus CBS 124228 (NR 172043.1)
Aspergillus microcysticus NRRL 4749 (NR 135401.1)
Aspergillus affinis ATCC MYA-4773 (NR 111684.1)
Aspergillus cretensis CBS 112802 (NR 135420.1)
Aspergillus westlandensis CBS 137321 (NR 135451.1)
Aspergillus neobridgeri NRRL 13078 (NR 135387.1)
Aspergillus westerdijkiae NRRL 3174 (NR 135389.1)
Aspergillus alliaceus NRRL 315 (NR 121331.1)
Aspergillus magaliesburgensis PPRI 6165 (NR 171610.1)
Pseudopenicillium giganteum CBS 144.69 (NR 160117.1)
Pseudopenicillium megasporum CBS 256.55 (NR 160081.1)
Penicillium estinogenum CBS 329.59 (NR 169981.1)
Penicillium oxalicum NRRL 787 (NR 121232.1)
Penicillium soosanum CCF 3778 (NR 173354.1)
Penicillium sp. MY2 (PP843229.1)
Penicillium bissettii CBS 140972 (NR 158821.1)
Penicillium araracuarense CBS 113149 (NR 119814.1)
Penicillium wotroi CBS 118171 (NR 119813.1)
Penicillium mariae-crucis CBS 271.83 (NR 111506.1)
Penicillium simplicissimum CBS 372.48 (NR 138290.1)
Penicillium cataractarum CBS 140974 (NR 158822.1)
Penicillium griseoflavum CGMCC 3.18799 (NR 173365.1)
Penicillium echinulonalgiovense CBS 328.59 (NR 172219.1)
Penicillium infrabuccalum DAOMC 250537 (NR 172032.1)
Penicillium pedernalense CSB 140770 (NR 146250.1)
Penicillium panissanguineum CBS 140989 (NR 158825.1)
Penicillium abidjanum CBS 246.67 (NR 111502.1)
Penicillium daleae CBS 211.28 (NR 111503.1)
Penicillium zonatum CBS 992.72 (NR 111501.1)
Penicillium brefeldianum NRRL 710 (NR 138263.1)
Penicillium limosum CBS 339.97 (NR 111496.1)
Penicillium lineolatum CBS 188.77 (NR 111500.1)
Penicillium annulatum CBS 135126 (NR 138303.1)
Penicillium rolfsii CBS 368.48 (NR 111669.1)
Penicillium subrubescens CBS 132785 (NR 111863.1)
Penicillium piscarium CBS 362.48 (NR 111507.1)
Penicillium guangxiense CGMCC 3.18793 (NR 173362.1)
Penicillium brasilianum CBS 253.55 (NR 111499.1)
Penicillium pulvillorum CBS 280.39 (NR 138292.1)
Penicillium ochrochloron CBS 357.48 (NR 111509.1)
Penicillium camponotum CBS 140982 (NR 158823.1)
Penicillium vasconiae CBS 339.79 (NR 138291.1)
Penicillium penarojense CBS 113178 (NR 138289.1)
Penicillium vanderhammenii CBS 126216 (NR 137577.1)
Penicillium amphipolaria CBS 140997 (NR 158827.1)
Penicillium tanzanicum CBS 140968 (NR 158820.1)
Penicillium reticulisporum NRRL 3447 (NR 121231.1)
Penicillium elleniae CBS 118135 (NR 138294.1)
Penicillium levitum CBS 345.48 (NR 111510.1)
Penicillium javanicum CBS 341.48 (NR 111511.1)
Penicillium oligosporum CBS 349.51 (NR 138330.1)
Penicillium caperatum CBS 443.75 (NR 138333.1)
Penicillium malacosphaerulum CBS 135120 (NR 138283.1)
Talaromyces aerugineus CBS 350.66 (NR 147420.1)
Talaromyces flavus CBS 310.38 (NR 147413.1)
Talaromyces viridis CBS 114.72 (NR 160136.1)

Figure S3. Phylogenetic placement of fungal strains UR1 and UR2 among next relates type strain material based on the ITS sequence of the rRNA operon. The depicted tree represents a neighbor joining tree which was calculated in MEGA11 considering 12390 nucleotide positions. Numbers at nodes represent bootstrap values of 70% and above (based on 100 replicate trees). Numbers in brackets are Acc numbers of respective sequence. Bar: Substitution per nucleotide position.

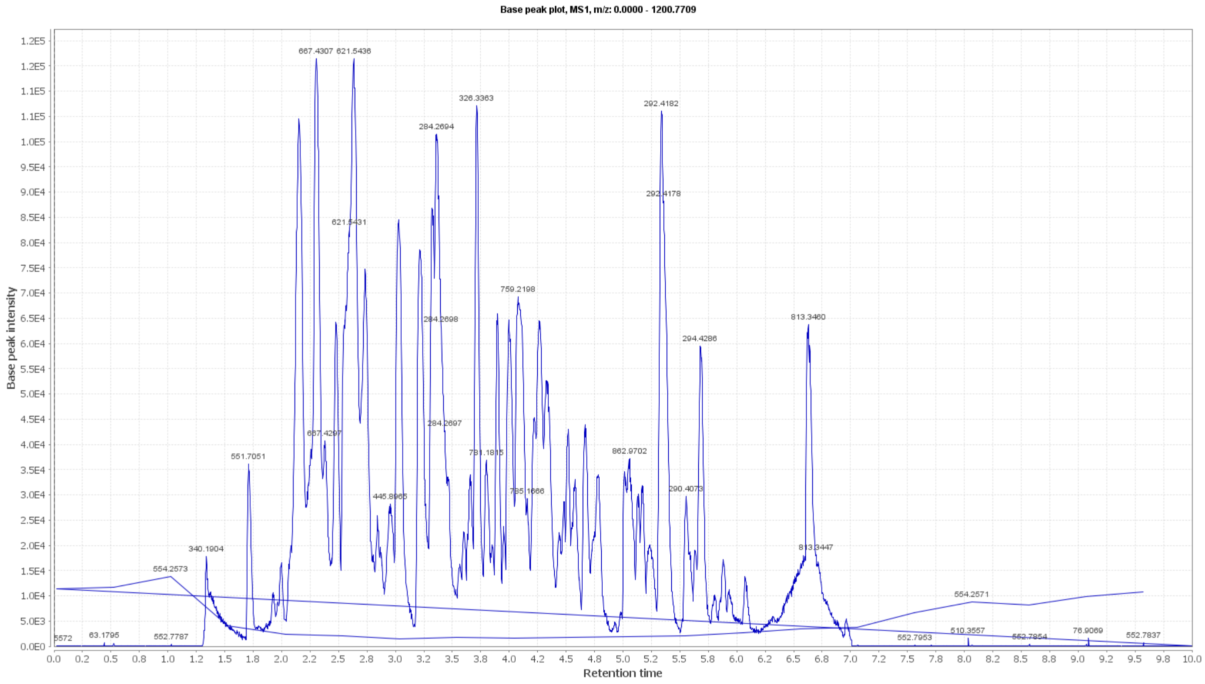

Figure S4. Total ion chromatogram of the marine sponge- associated *Aspergillus* sp. (UR1) total extract in negative ionization mode

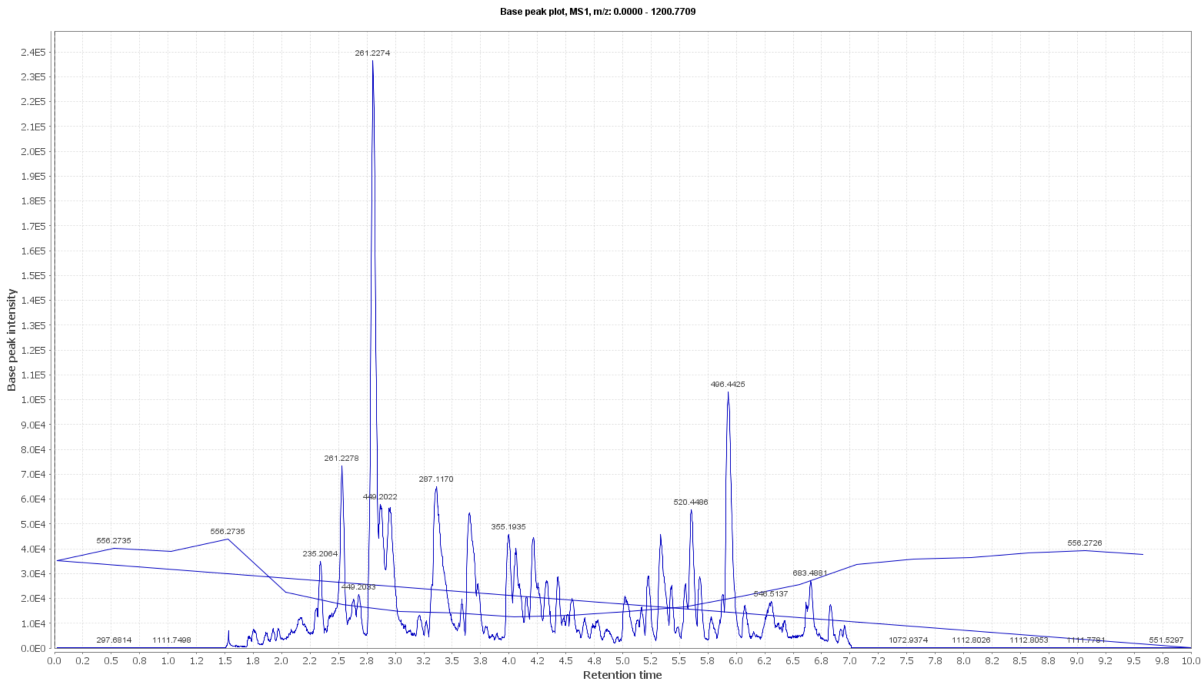

Figure S5. Total ion chromatogram of the marine sponge- associated *Aspergillus* sp. (UR1) total extract in positive ionization mode

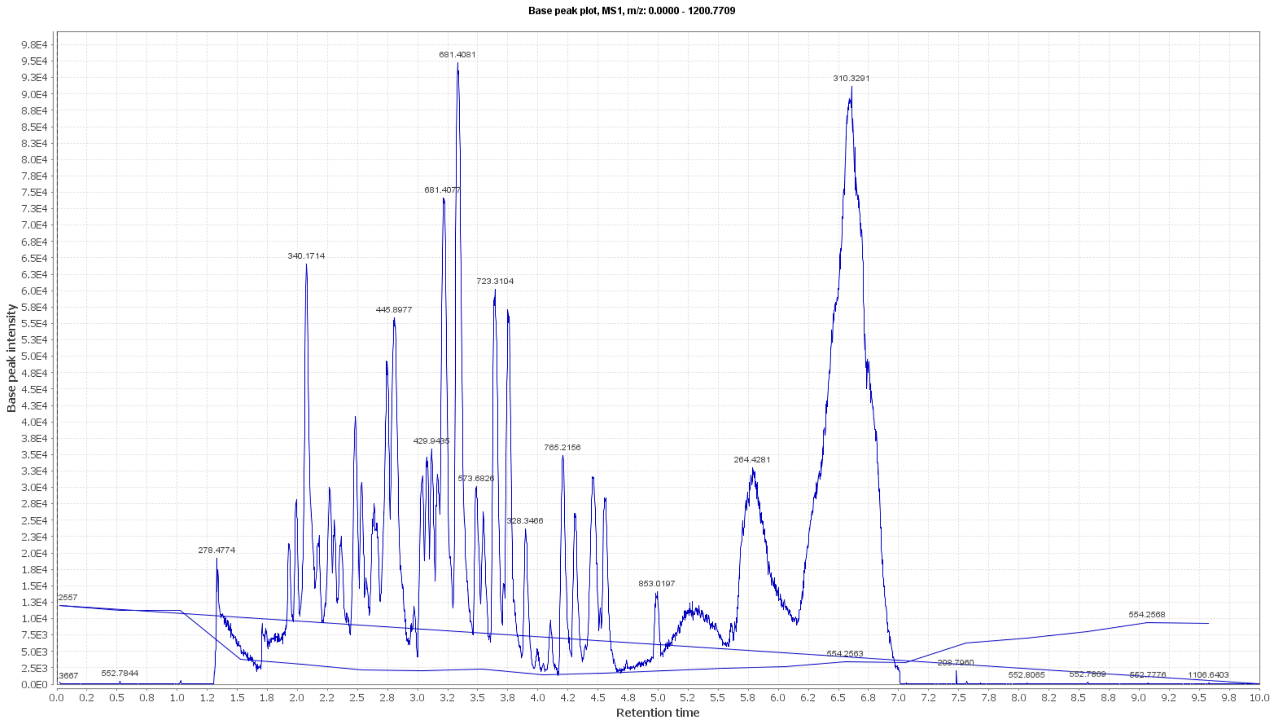

Figure S6. Total ion chromatogram of the marine sponge- associated *Penicillium* sp. (UR2) total extract in negative ionization mode

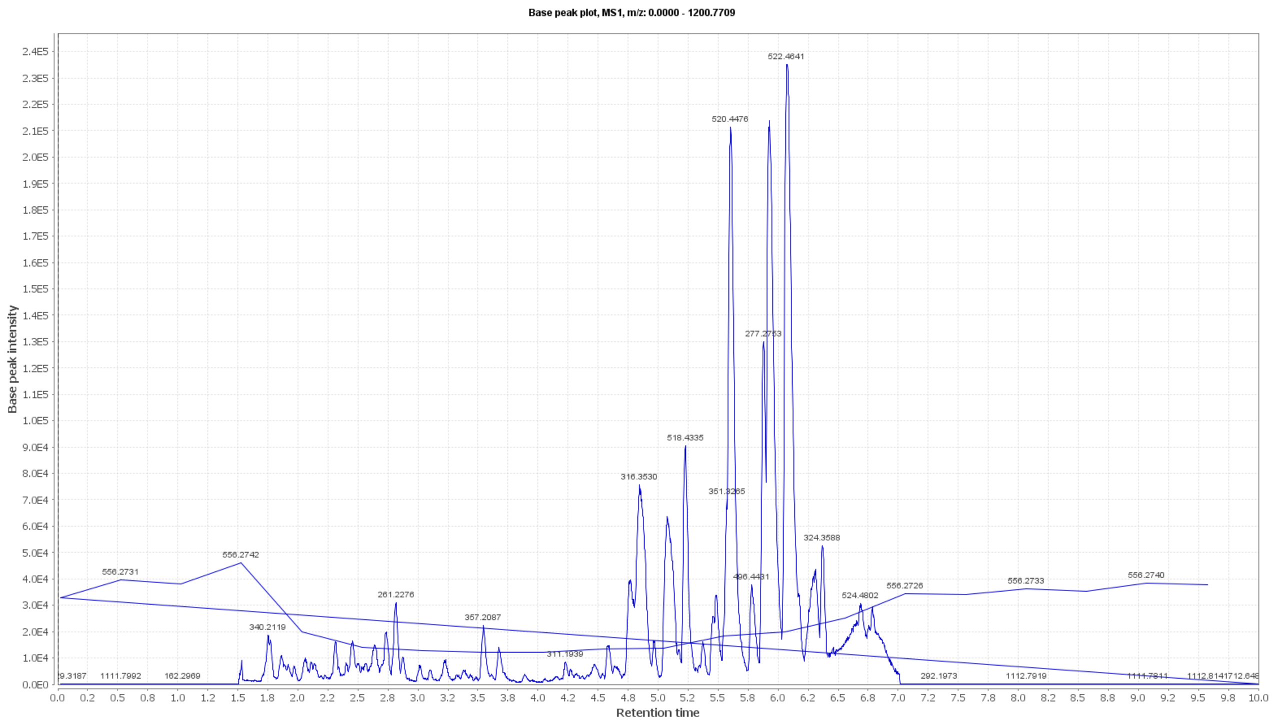

Figure S7. Total ion chromatogram of the marine sponge- associated *Penicillium* sp. (UR2) total extract in positive ionization mode

Table S1. Dereplication of secondary metabolites detected in extracts of *Spheciospongia vagabunda*-derived fungi *Aspergillus* sp. (UR1) and *Penicillium* sp. (UR2)

Fungal strain	No.	Rt (min)	*m/z* (ionization)	Molecular weight	Molecular formula	Putative identification	Chemical class	Source
***Aspergillus* sp. (UR1)**	**1**	4.17	563.3117 $[M+H]^+$	562.3045	$C_{33}H_{42}N_2O_6$	Teraspiridole A	Alkaloid	Creek-bottom-derived *Aspergillus terreus*
	**2**	5.81	376.1298 $[M-H]^-$	377.1371	$C_{21}H_{19}N_3O_4$	Circumdatin J	Alkaloid	Marine fungus *Aspergillus ostianus*
	**3**	5.86	318.2800 $[M+H]^+$	317.2728	$C_{21}H_{35}NO$	Asperidine B	Alkaloid	Soil-derived *Aspergillus sclerotiorum* PSU-RSPG178
	**4**					Preussin	Alkaloid	Fungus *Simplicillium lanosoniveum* TAMA 173
	**5**	4.55	447.2957 $[M+H]^+$	446.2885	$C_{24}H_{38}N_4O_4$	Sclerotiotide C	Cyclic peptide	Salt sediment-derived *Aspergillus sclerotium* PT06-1
	**6**	3.59	465.2145 $[M+H]^+$	464.2072	$C_{25}H_{28}N_4O_5$	Aspercolorin	Cyclic peptide	*Aspergillus versicolor*
	**7**	4.70	407.2345 $[M+H]^+$	406.2272	$C_{24}H_{35}ClO_3$	ICM0301 C	Polyketide	*Aspergillus* sp. F-1491
	**8**	5.21	312.1804 $[M+H]^+$	311.1732	$C_{16}H_{25}NO_5$	Wasabidienone E	Polyketide	Sponge-derived *Aspergillus flocculosus* 01nt.1.1.5
	**9**	4.01	337.1789 $[M+H]^+$	336.1716	$C_{22}H_{24}O_3$	Asperrubrol	Phenylpolyene	*Aspergillus niger*
	**10**	5.36	414.1562 $[M-H]^-$	415.1635	$C_{22}H_{25}NO_7$	14-Hydroxy-6β-*p*-nitrobenzoyl-cinnamolide	Sesquiterpenoid nitobenzoyl estes	Marine green alga-derived *Aspergillus versicolor*
	**11**	1.96	321.2429 $[M+H]^+$	320.2357	$C_{20}H_{32}O_3$	Aspergiloid D (Isopimarane)	Diterpenoids	Endophytic *Aspergillus* sp. YXf3

	**12**	2.13	396.1078 [M-H]$^-$	397.1151	$C_{21}H_{19}NO_7$	Azaspirofuran B	Hetero-spirocyclic γ-lactam	Marine sediment-derived *Aspergillus sydowii* D2-6
	**13**	1.78	231.1346 [M+H]$^+$	230.1273	$C_{10}H_{18}N_2O_4$	Terramide C	Piperazine-2,5-dione (cyclic *bis*-hydroxamic acids)	*Aspergillus terreus* CMI 44339
***Penicillium* sp. (UR2)**	**14**	2.08	368.2323 [M+H]$^+$	367.2250	$C_{22}H_{29}N_3O_2$	Brevicompanine A	Alkaloid	*Penicillium brevicompactum*
	**15**					*allo*-Brevicompanine B		Deep-ocean sediment-derived *Penicillium* sp.
	**16**	4.64	397.2118 [M+H]$^+$	396.2045	$C_{23}H_{28}N_2O_4$	Mollenine B	Alkaloid	*Eupenicillium molle* NRRL 13062
	**17**	3.20	397.2476 [M+H]$^+$	396.2404	$C_{24}H_{32}N_2O_3$	TAN-1251B	Alkaloid	*Penicillium thomii* RA-89
	**18**	1.96	321.2429 [M+H]$^+$	320.2357	$C_{20}H_{32}O_3$	Conidiogenone H	Diterpene	Marine red alga-derived *Penicillium chrysogenum* QEN-24S
	**19**	3.63	439.2483 [M+H]$^+$	438.2411	$C_{27}H_{34}O_5$	Brevione I	Spiroditerpenoid	Deep sea sediment-derived *Penicillium* sp.
	**20**	3.63	439.2483 [M+H]$^+$	438.2411	$C_{27}H_{34}O_5$	Arisugacin F	Meroterpenoid	Endophytic fungus *Penicillium* sp. SXH-65
	**21**	3.06	519.2962 [M+H]$^+$	518.2889	$C_{29}H_{42}O_8$	Austalide H acid butyl ester	Meroterpenoid	Marine brown alga-derived *Penicillium thomii* KMM 4645
	**22**	3.17	455.2285 [M+H]$^+$	454.2212	$C_{24}H_{30}N_4O_5$	Viridic acid	Tetrapeptide	*Penicillium viridicatum*
	**23**	2.97	323.1845 [M+H]$^+$	322.1773	$C_{18}H_{26}O_5$	Rezishanone D	Polyketide	*Penicillium notatum*

	**24**	2.05	325.2179 [M-H]$^-$	326.2251	$C_{22}H_{30}O_2$	Penostatin C	Benzopyran	Marine green alga-derived *Penicillium* sp.
	**25**	4.50	211.1323 [M+H]$^+$	210.1250	$C_{12}H_{18}O_3$	Patulolide A	Macrolide	*Penicillium urticae* SIIR59
	**26**	4.62	386.2335 [M-H]$^-$	387.2408	$C_{23}H_{33}NO_4$	Cissetin	Methyl-pyrrolidine-2,4-dione	Endophytic fungus *Preussia* sp.

Table S2. Topological parameters of top 10 hub genes

No.	Name	Target	Degree	Betweenness	Closeness
**1**	Epidermal growth Factor	EGFR	19	0.0851	1
**2**	Peroxisome proliferator-activated receptor gamma	PPARG	18	0.0670	0.9500
**3**	Estrogen receptor alpha	ESR1	17	0.0328	0.9048
**4**	Glycogen synthase kinase-3 beta	GSK3B	17	0.0354	0.9048
**5**	Serine/threonine-protein kinase	MTOR	16	0.0303	0.8636
**6**	Poly [ADP-ribose] polymerase 1	PARP1	16	0.0296	0.8636
**7**	Matrix metalloproteinase-9	MMP9	15	0.0179	0.8261
**8**	G1/S-specific cyclin-D1	CCND1	15	0.0161	0.8261
**9**	Androgen receptor	AR	14	0.0157	0.7917
**10**	Prostaglandin G/H synthase 2	PTGS2	14	0.0110	0.7917

Table S3. Docking scores of tested compounds

Fungal strain	Cpd	Docking Score (kcal/mol)			
		EGFR (1M17)	PPARG (7AWD)	ESR1 (7UJW)	GSK3B (5K5N)
Aspergillus sp. (UR1)	**1**	-3.86	**ND**	**ND**	-5.21
	**2**	-5.75	-7.85	**ND**	-5.95
	**3**	-3.84	-6.77	-5.18	-4.70
	**4**	-4.16	-6.44	-5.15	-4.56
	**5**	-3.83	**ND**	-3.78	-3.19
	**6**	-6.18	-9.13	-1.82	-5.78
	**7**	-4.62	-6.35	-7.86	-5.77
	**8**	-5.20	-6.80	-6.08	-4.97
	**9**	**ND*	-7.22	**ND**	**ND**
	**10**	-3.85	-7.27	-7.58	-3.59
	**11**	-4.62	-7.01	-6.93	-5.76
	**12**	-5.43	-8.13	-4.58	-5.28
	**13**	-6.27	-6.66	-7.44	-5.61
Penicillium sp. (UR2)	**14**	-3.69	-6.96	-7.60	-5.14
	**15**	-4.81	-7.08	-8.21	-4.55
	**16**	-4.15	-8.38	**ND**	-5.01
	**17**	-5.52	-8.04	-6.58	-6.39
	**18**	-4.93	-6.76	-6.99	-5.71
	**19**	-3.58	**ND**	**ND**	-3.93
	**20**	**ND**	**ND**	**ND**	-4.42
	**21**	-6.23	-7.06	-4.69	**ND**
	**22**	0.22	-6.76	**ND**	**ND**
	**23**	-4.05	-5.79	-5.31	-5.09
	**24**	-5.11	-7.88	-5.78	-5.16
	**25**	-4.87	-6.66	-7.31	-6.76
	**26**	-4.51	-6.88	-5.73	-4.75
	**Co-crystalized Ligand**	**-7.90 RMSD= 1.18 A°**	**-10.94 RMSD= 0.59 A°**	**-11.58 RMSD= 1.05 A°**	**-8.26 RMSD= 0.76 A°**
